# The Effect of Protracted Exposure to Radiation on Liver Injury: A Cohort Study of Industrial Radiographers in Xinjiang, China

**DOI:** 10.3390/ijerph15010071

**Published:** 2018-01-04

**Authors:** Qing Sun, Weiming Mao, Haiyue Jiang, Xiaoyue Zhang, Jing Xiao, Yulong Lian

**Affiliations:** Division of Occupational and Environmental Health, College of Public Health, Nantong University, Se Yuan Road, No. 8, Nantong 226019, China; SunQ_9005@163.com (Q.S.); mwm457419340@163.com (W.M.); echo_jhy@163.com (H.J.); m18862920791@163.com (X.Z.); Xiaoj_1980@163.com (J.X.)

**Keywords:** liver injury, cohort studies, occupational exposure, radiation, risk factors

## Abstract

Background: At present, a large number of studies indicate that high dose ionizing radiation exposure is an important risk factor for liver damage. Whether protracted exposure to low external doses of ionizing radiation could induce liver injury is unclear. The aim of this study was to assess the risk of liver injury following protracted exposure to occupational radiation compared to a group of unexposed workers. Methods: A three-year cohort study was initiated in Xinjiang, China in 2010 and included 508 industrial radiographers and 2156 unexposed workers. The incidence of liver injury was assessed clinically based on the evaluation of alanine aminotransferase (ALT) and aspartate transaminase (AST) levels. Logistic regression was used to examine whether radiation is a risk factor for liver injury. Results: Compared with the unexposed group, protracted radiation exposure was found to be a risk factor for liver injury. Sex, age at baseline and alcohol consumption were not associated with liver injury. However, hypertension was a risk factor for liver injury. The association between cumulative recorded radiation dose and liver injury was not found in this study. Conclusions: These findings indicate that protracted exposure to radiation is a risk factor for liver injury.

## 1. Introduction

Radiologic technology, a non-destructive technique [1,2], has been widely used in many fields, such as agriculture, medical treatment and the manufacturing industry. With the development of science and technology, the number of industrial radiographers has quickly increased in recent years and is one of the most important populations exposed to ionizing radiation. It has been reported that ionizing radiation exposure can induce biological effects on human health [3], with both early and late effects of radiation in normal tissues and organs [3,4]. Previous studies on patients without liver disease who undergo radiation therapy have proved that exposure to ionizing radiation can induce hepatic dysfunction or even liver cancer [5,6,7,8]. Data from cohorts of atomic bomb survivors indicate that exposure to radiation induced a high prevalence of hepatitis B and hepatitis C infection [9] and liver cirrhosis [6,9]. Several studies have shown that acute high dose radiation (>10 Gy) can cause significant structural damage in the liver and hepatic toxicity [10]. Experiments carried out on rats showed that systemic exposure to a single dose of 6 Gy gamma radiation could induce a significant elevation in alanine aminotransferase (ALT) [11]. An epidemiology study on radiotherapy of cancer in childhood and adolescence indicated that radiation of the liver (mean liver dose = 5 Gy) could cause low hepatotoxicity [12]. The studies mentioned above indicate that exposure to high dose ionizing radiation can induce liver damage. However, the International Commission on Radiological Protection (ICRP) recommended that the annual effective dose limits for radiation workers should no more than 20 mSv, and this criterion has been implemented in most countries [13]. The effects of protracted exposure to this low external dose of ionizing radiation on liver injury is not clear. Researches have shown that liver is considered a radiation sensitive organ [12,14]. Animal experimental data indicate that low dose radiation (single dose ranging from 0.02 to 1.0 Gy) can increase liver inflammation [15], which suggests that low dose radiation exposure may negatively affect liver function and result in the development of liver disease.

The aim of this study was to evaluate the relationship between protracted exposure to low dose radiation and liver injury in industrial radiographers, and complemented the current risk estimates that mainly focus on high-dose or single-dose radiation exposure-induced liver injury. All of the industrial radiographers predominantly exposed to X-ray and γ-ray radiation, and their liver function were assessed each year during the follow-up period. After adjusting for other liver injury confounding risk factors, this study estimated the risk of liver injury from low external doses of ionizing radiation exposure, compared with the unexposed group.

## 2. Materials and Methods

### 2.1. Study Population and Follow Up

The population in this study consisted of petrochemical workers who were registered at the Karamay Center for Disease Control (CDC, Karamay, China). All the radiographers included in this cohort were predominantly exposed to external X-ray and γ-ray radiation and the dose was below the dose limit of the Protection of Ionizing Radiation and Basic Safety Standard of Radiation Source in China, 20 mSV. When a radiographer’s radiation dose exceeded the limit, the worker was removed from his position. In 2010, baseline data of medical examination results and baseline questionnaire for 766 radiographers and 3610 control workers were obtained. The contents of baseline questionnaire include social demographic characteristics and lifestyle factors such as sex, age at baseline, education level, and marital status, history of cigarette smoking and alcohol consumption. In all, 97 radiographers and 940 non-exposed workers were excluded because of loss to follow-up, had a history of liver disease and worked for less than one year at baseline. 145 radiographers and 223 non-exposed workers were also excluded because of elevated ALT and aspartate transaminase (AST) at baseline. Furthermore, participants with viral hepatitis (n = 46), fatty liver disease (n = 34), cholecystitis (n = 0), gallstones (n = 208) were also excluded. The final analytic cohort was comprised of 2683 participants including 508 exposed workers and 2175 non-exposed workers. In addition to the social demographic characteristics mentioned above, other possible risk factors that could induce liver injury including hypertension and diabetes were also assessed during the baseline period according to a self-reported questionnaire and occupational health examination database. Fatty liver, cholecystitis and intrahepatic gallstones diagnosed annually by an abdominal ultrasound examination during the baseline and follow up period. Follow-up was from January 2010 to liver injury diagnosis, loss of follow up, retirement or 31 December 2013. During the follow-up period, annual occupational health examinations were conducted in Karamay CDC, and all participants attended the examination. According to the rules of the People’s Republic of China on the prevention and control of occupational diseases, new cases with liver injury were identified. The research protocol for this cohort study was approved annually by the ethics committee of Xinjiang Medical University. All participants enrolled in the study provided written informed consent.

### 2.2. Clinical Evaluation of Liver Injury

Annual occupational health examinations were conducted in the medical center at the Department of Occupational Health, Karamay CDC. All workers had a complete physical examination as well as venous blood collection, which were used to evaluate the ALT and AST levels. The workers also complete the abdominal ultrasound examination, an important way to detect possible abnormal liver morphology. Liver enzyme activity and other test results were compared to normal laboratory standards of Karamay CDC.

Previous studies have suggested that serum ALT and AST are sensitive and reliable indicators of liver disease [16], and can satisfy most of the criteria for a screening test [17]. We accepted the upper limit of normal (ULN) value of ALT and AST as 40 IU/L (IU Refers to the amount of enzyme that converts 1 micromolar substrate per minute under specific conditions or the amount of enzyme that converts 1 micromolar related moiety in the substrate) [18,19]. Because of the criterion of define liver injury around the world are still controversial, and the definition of radiation induced liver injury is unclear. The Asian-Pacific clinical practice guidelines believed that serum ALT level is raised if it is more than twice ULN, and previous USA studies recommended that the concentration of ALT or AST more than twice normal was the criterion of toxin-induced hepatic injury [20,21,22]. So, we use this criterion to define liver injury in our study.

### 2.3. Radiation Dosimetry

The consistent dosimetric instrument, thermoluminescent dosimeter (TLD), was used to monitor the exposure to X-rays and γ-rays of the industrial radiographers, who were registered at the Karamay CDC. Each of the radiographers was provided one TLD, and worn it on the right chest over the lead protective garment. Based on the TLD, we get the radiation dose record, and calculate dose after upward adjustment, using the apron attenuation factor, if the badge was reported to have been worn under the apron. Adjusted for the use of protective lead aprons, the estimated absorbed doses of liver will be more accurate.

Job exposure matrix (JEM) is a common occupational risk assessment method [23,24], that can be used to assess the contribution of occupationally exposed occupational risk factors for the disease [23]. Due to the loss of some of the individual test data in previous years, we use the average dose of other research years of the same work type to supplement the missing data, and evaluate the cumulative radiation dose based on the JEM. Due to some workers engaged in radiation work only a few months, and it is difficult to estimate their first year’s radiation exposure dose. So, we exclude workers with a working history of less than 1 year. The cumulative radiation dose was calculated according to the following equation:

$$D=\overset{n}{\underset{i=1}{\sum }}X_{i}$$

D is cumulative radiation exposure dose (mSv), $X_{i}$ is the radiation exposure dose in the *i*-th year of the industrial radiographers engaged in radiological work.

### 2.4. Confounding Factors

Several factors were considered as potential confounders which were extracted from baseline data and defined before we analyze the liver injury. Age was categorized into less than 30 years old, 30-year, 40-year and 50-year. Education was categorized into junior high, high school and college. Information regarding smoking and alcohol consumption were also collected. Alcohol consumption was based on the question: ’Do you drink more recently?’ The response options were never, seldom, sometimes, often and always. We categorized the response ‘never’ into never alcohol consumption, ‘seldom’ and ‘sometimes’ into alcohol consumption occasionally, ‘often’ and ‘always’ into alcohol consumption frequently [25]. Smoking was based on the question: ’Do you smoke more recently?’ The response options were never, occasionally and frequently [26]. World health organization (WHO) recommended that the hypertension definition was systolic blood pressure greater than or equal to 140 mmHg and/or diastolic blood pressure greater than or equal to 90 mmHg. WHO also defined diabetes as fasting plasma glucose value ≥7 mmol/L [27]. We categorized hypertension and diabetes according to this definition.

### 2.5. Statistical Methods

The demographic and clinical variables of industrial radiographers and control workers were described and compared using the chi-square test. We calculated risk ratios (RR) and 95% confidence intervals (CI) with unadjusted logistic regression model and multiple logistic regression model to analyze the effects of protracted low-dose radiation exposure on liver injury. The multiple model was adjusted for sex, education, age at baseline, marital status, smoking, alcohol consumption, hypertension and diabetes.

To evaluate the independent effects of each confounding factor on the incidence of liver injury among industrial radiographers, we presented crude and adjusted multivariate model. The multivariate model included the following baseline values: sex, age at baseline, marital status, education level, smoking, alcohol consumption, hypertension, diabetes and cumulative recorded radiation adjustment. The unadjusted model showed the single risk factors. The cumulative radiation dose was calculated according to JEM mentioned above. The relationship between the cumulative radiation dose and the relative risk for liver injury was investigated by trend analysis, using three equal dose categories (>0–5.167, 5.168–31.540, 31.541–110.835 mSv).

## 3. Results

In this study, 508 industrial radiographers 2175 non-exposed workers were analyzed. The fluctuation range of ALT was 0–301 U/L, and AST was 2–106 U/L. Table 1 describes the characteristics of the industrial radiographers and the unexposed worker group. The total number of participants in this study was divided into four groups according to age at baseline (age less than 30 years old, 30-year, 40-year, 50-year). The mean age of the industrial radiographers at baseline was 36.74 years (range, 20–65 years), and 89.53% were male. In the unexposed workers, the mean age at baseline was 38.25 years (range, 18–59 years), and 61.35% were male. The frequency of smoking and high school education level in industrial radiographers were higher than those in the unexposed group. Sex and age at baseline, marital status, education level, alcohol consumption, hypertension, diabetes and gallstones were significantly different between the industrial radiographers and the unexposed group.

The prevalence, unadjusted and adjusted logistic RR, and 95% CIs of liver injury are shown in Table 2. The incidence of liver injury in the low-dose radiation exposure group was approximately two to three times more frequent than that in the unexposed group. In the unadjusted analysis, the radiation exposure group had a total RR of 2.642 (95% CI 1.850 to 3.772) compared with the non-radiation exposure group. However, after adjusting the confounders, the total RR decreased to 2.227 (95% CI 1.526 to 3.252). Both the unadjusted and adjusted analyses showed that after differentiating sex, age at baseline, marital status, education level, smoking and alcohol consumption, the associations of protracted low-dose radiation exposure and liver injury remained significant among males, age at baseline between 30 and 50 years old, those who married/living with a partner, those who received higher education (high school and college), those who never smoked and smoking/drinking occasionally and frequently. Particularly, in the unadjusted analysis, protracted low-dose radiation exposure is not a risk factor for liver injury in females. After adjustment of the confounders, this association became significant. However, in the unadjusted analysis, protracted low-dose radiation exposure is a risk factor for liver injury in those who never consumed alcohol and singles; when we adjust the potential confounders, this association became non-significant.

In the industrial radiographers, logistic regression was used to estimate the risk factors associated with the incidence of liver injury. After adjusting for basic social demographic characteristics and all possible confounding risk factors, two models are shown in Table 3. The unadjusted model and adjusted model indicated that hypertension was a significant risk factor for liver injury. In addition, increased cumulative radiation dose and age at baseline (*p* > 0.05) were not associated with increased risk of liver injury.

## 4. Discussion

To our knowledge, this is the first evaluation of the relationship between long-term exposure to low-dose ionizing radiation below the threshold limit value (TLV) and liver injury in industrial radiographers compared with a non-exposed group. This study complements the current risk estimates that mainly focus on high-dose or single-dose radiation exposure-induced liver injury in other studies. We found that protracted exposure to low-dose radiation was associated with liver injury. Therefore, the present study provides direct estimates of the risk of liver injury caused by protracted occupational exposure to low-dose radiation. Previous research has reported that the liver is a radiation-sensitive organ [12,14]. We found that protracted low-dose radiation exposure was a risk factor for liver injury.

We also found that sex and age were not associated with liver injury, which was in agreement with the longitudinal data collected from the atomic bomb survivors, which suggested that sex [8,9] and age [9] were not associated with radiation-induced liver injury. Evidence from epidemiology studies of the general population also suggested that there was no association between age, serum ALT level [28] and liver injury [29].

Our results showed that hypertension is a risk factor for liver injury and were in agreement with previous study findings [30]. Although it is generally known that excessive alcohol consumption is a risk factor for liver disease [31,32], previous studies have confirmed that light [33] or modest alcohol consumption [34] may be a protective factor in liver injury. After adjusting for covariates, our data clearly indicated that alcohol consumption was not a risk factor. Given that the daily alcohol consumption in participants was less than medium drinking defined in previous studies [33,35], our results are in agreement with those in previous studies [33,35].

No relationship between cumulative radiation dose and liver injury was found in the present study. This disagreed with the results of previous studies following exposure to high doses of radiation, which suggested that exposure to higher cumulative radiation doses results in a greater increase in ALT [36], relative risk of liver disease [5,7,9] and the severity of liver disease caused by radiation is related to the absorbed dose [37]. The observed divergence in liver function may be due to liver tolerance to ionizing radiation [36]. The total dose required to induce a tissue effect (i.e., the tolerance dose) increases if exposure occurs in smaller dose fractions or at smaller dose rates [4]. The liver has a special architecture, which allows it to tolerate a substantially higher dose of radiation prior to clinical manifestations [7]. A previous study established a tolerance level with a mean dose of <30–32 Gy (TD5) and <42 Gy (TD50) in whole-liver irradiation [38]. However, the radiographers in our study were exposed to low-dose radiation, and the follow-up period in our cohort was not long enough. The studies on atomic bomb survivors who were exposed to high-dose radiation in Japan suggested that persistent HBV and HCV infections were associated with the dose-related increase in the incidence of chronic liver disease and cirrhosis [9]. However, additional pathological studies demonstrated that atomic bomb radiation did not increase the risk of liver cirrhosis, and the prevalence of anti-HBV surface antigen and anti-HCV antibody increased in the high-dose subjects [39], but not in subjects exposed to low-dose radiation [6]. This phenomenon may explain the results in the present study.

Direct estimates of the risk of liver injury due to protracted radiation exposure, compared with the unexposed group, is a strength of the present cohort study. According to the radiation protection requirements of our country, all the radiographers were required to wear radiation protection equipment during their employment. Because of the sufficient protection equipment and protection time, we did not analyze the impact of protection. There were many disequilibria between the exposed and control groups, so this study used a stratified analysis method to compensate for the deficiency. However, there are also limitations to this study. First, some of the individual test data were missing in previous years in this study, the cumulative radiation dose of the radiographers evaluated according to the job exposure matrix may cause measurement bias, and the individual radiation dose should be collected for further study. Second, we divided the participants into three groups based on the frequency of alcohol consumption (never, occasionally and frequently), and did not get the information of alcohol drinking volume, which may have led to bias. Finally, several other potential confounders were not examined in the study, including socioeconomic status, medications, diet and level of physical activity and exercise, which might have contributed to an under- or overestimation of the associations. However, the socioeconomic status and lifestyle were similar because the control and exposed groups lived in the same city and had the same company, thus we believe that the bias might be minimal. In addition, because the follow-up period was short and the cohort size small, it is possible that some statistically significant findings may be due to chance.

## 5. Conclusions

The present study is unique in that the risk of liver injury due to protracted exposure to low-dose radiation was estimated. The results demonstrated that protracted low-dose radiation exposure is a risk factor for liver injury. The results of the present study provide a basis for further studies on the effects of protracted low-dose radiation exposure on the liver and other systems. The societal and public health significance of the early detection and prevention of protracted low dose radiation induced occupational population hepatic injury in occupational environment is emphasized in this study.

## Figures and Tables

**Table 1:** Characteristics of the study population.

Variables	Unexposed Group (n = 2175)	Industry Radiographer (n = 508)	*p* Value
Sex			
Male	1407 (64.69)	454 (89.37)	
Female	768 (35.31)	54 (10.63)	0.0001
Age at baseline			
<30	357 (16.41)	144 (28.35)	
30-	771 (35.45)	137 (26.97)	
40-	925 (42.53)	194 (38.19)	
50-	122 (5.61)	33 (6.50)	0.0001
Marital status			
Single	370 (17.01)	108 (21.26)	
Married/living with a partner	1072 (49.29)	255 (50.20)	
Divorced	207 (9.52)	35 (6.89)	
Widowed	526 (24.18)	110 (21.65)	0.037
Education level			
Junior high	150 (6.90)	39 (7.68)	
High school	859 (39.49)	265 (52.17)	
College	1166 (53.61)	204 (40.16)	0.0001
Smoking			
Never	1340 (61.61)	284 (55.91)	
Occasionally	216 (9.93)	82 (16.14)	
Current	619 (28.46)	142 (27.95)	0.0001
Alcohol consumption			
Never	445 (20.46)	154 (30.31)	
Occasionally	1172 (49.29)	208 (40.94)	
Current	658 (30.25)	146 (28.74)	0.0001
Hypertension			
No	1580 (72.64)	401 (78.94)	
Yes	595 (27.36)	107 (21.06)	0.004
Diabetes			
No	2157 (99.17)	507 (99.80)	
Yes	18 (0.83)	1 (0.20)	0.233
Cumulative recorded radiation dose			
0	2175 (100)		
>0–5.167		169	
5.168–31.540		174	
31.541–110.835		165	

**Table 2:** Logistic regression RR and 95% CI of Liver injury in those exposed to radiation compared with unexposed workers.

Variables	Non-Radiation Exposure	Radiation Exposure	Un-Adjusted		Adjusted *	
	Cases/Total	Cases/Total	RR	95% CI	RR	95% CI
**Total**	90/2175	52/508	2.642	1.850–3.772 ^a^	2.227	1.526–3.252 ^a^
Sex						
Male	69/1407	48/454	2.293	1.560–3.368 ^a^	2.161	1.447–3.227 ^a^
Female	21/768	4/54	2.846	0.941–8.608	3.319	1.062–10.37 ^a^
Age at baseline						
<30	27/357	18/144	1.746	0.929–3.281	1.562	0.805–3.029
30-	31/771	16/137	3.156	1.676–5.946 ^a^	2.780	1.402–5.512 ^a^
40-	26/925	15/194	2.898	1.504–5.580 ^a^	2.835	1.404–5.724 ^a^
50-	6/122	3/33	1.933	0.457–8.184	1.976	0.408–9.564
Marital status						
Single	25/370	15/108	2.226	1.128–4.393 ^a^	1.973	0.935–4.166
Married/living with a partner	36/1072	25/255	3.128	1.841–5.314 ^a^	2.855	1.639–5.078 ^a^
Divorced	6/207	2/35	2.030	0.393–10.489	1.702	0.277–10.449
Widowed	23/526	10/110	2.187	1.010–4.737 ^a^	2.018	0.892–4.566
Education level						
Junior high	6/150	3/39	2.000	0.477–8.384	1.437	0.287–7.192
High school	36/859	25/265	2.381	1.401–4.046 ^a^	2.134	1.215–3.746 ^a^
College	48/1166	24/204	3.106	1.856–5.196 ^a^	2.462	1.411–4.297 ^a^
Smoking						
Never	54/1340	24/284	2.198	1.335–3.621 ^a^	1.865	1.090–3.192 ^a^
Occasionally	2/216	10/82	14.861	3.181–69.424 ^a^	15.634	2.611–93.620 ^a^
Frequently	34/619	18/142	2.498	1.366–4.566 ^a^	2.086	1.105–3.938 ^a^
Alcohol consumption						
Never	19/445	16/154	2.600	1.301–5.194 ^a^	2.049	0.976–4.303
Occasionally	41/1072	19/208	2.528	1.436–4.451 ^a^	2.342	1.283–4.275 ^a^
Frequently	30/658	17/146	2.759	1.477–5.151 ^a^	2.238	1.168–4.288 ^a^

* Adjusted for sex, age at baseline, marital status, education level, smoking, alcohol consumption, hypertension, diabetes; ^a^ *p* < 0.05.

**Table 3:** Logistic regression RR and 95%CI of liver injury in industrial radiographers.

Covariate	Liver Injury Cases/Total Participants	Model 1		Model 2	
		RR	95% CI	RR	95% CI
Sex					
Male	48/454	1.0		1.0	
Female	4/54	0.677	0.234–1.956	0.949	0.300–3.001
Age at baseline					
<30	18/144	1.0		1.0	
30-	16/137	0.926	0.451–1.898	1.192	0.486–2.924
40-	15/194	0.587	0.285–1.208	0.531	0.160–1.759
50-	3/33	0.700	0.194–2.531	0.666	0.115–3.862
Marital status					
Single	15/108	1.0		1.0	
Married/living with a partner	25/255	0.674	0.340–1.335	0.993	0.384–2.566
Divorced	2/35	0.376	0.082–1.732	0.597	0.103–3.452
Widowed	10/110	0.620	0.265–1.448	0.593	0.204–1.727
Education level					
Junior high	3/39	1.0		1.0	
High school	25/265	1.250	0.359–4.353	1.309	0.344–4.974
College	24/204	1.600	0.457–5.598	1.397	0.336–5.811
Smoking					
Never	24/284	1.0		1.0	
Occasionally	10/82	1.505	0.688–3.291	1.432	0.630–3.254
Frequently	18/142	1.573	0.823–3.005	1.248	0.625–2.492
Alcohol consumption					
Never	16/154	1.0		1.0	
Occasionally	19/208	0.867	0.430–1.747	1.016	0.483–2.135
Frequently	17/146	1.137	0.551–2.344	1.394	0.547–3.553
Hypertension					
No	35/401	1.0		1.0	
Yes	17/107	1.975	1.059–3.685 ^a^	2.286	1.143–4.572 ^a^
Cumulative recorded radiation dose					
>0–5.167	22/169	1.0		1.0	
5.168–31.540	14/174	0.585	0.288–1.185	0.635	0.271–1.488
31.541–110.835	16/165	0.718	0.362–1.421	0.998	0.330–3.020

Model 1: unadjusted; Model 2: Adjusted for sex, age at baseline, marital status, education level, smoking, alcohol consumption, cumulative recorded radiation dose, hypertension, diabetes; ^a^ *p* < 0.05.

## Abbreviations

The following abbreviations are used in this manuscript:

ALT	Alanine aminotransferase
AST	Aspartate transaminase
CDC	Center for Disease Control
ICRP	The International Commission on Radiological Protection
JEM	Job exposure matrix
TLD	Thermoluminescent dosimeter
TLV	The threshold limit value
ULN	The upper limit of normal
WHO	World health organization
